# Comparison of clinical independence level scores among predoctoral dental students between dental school clinic and community clinic rotation

**DOI:** 10.1002/jdd.13803

**Published:** 2024-12-18

**Authors:** Anubhuti Shukla, Vaishnavi Amrutham, Amanda Albright

**Affiliations:** ^1^ Department of Dental Public Health and Dental Informatics Indiana University School of Dentistry Indianapolis Indiana USA

**Keywords:** community‐based dental education, curriculum development/evaluation, dental education, evaluation of clinical performance, predoctoral dental education

## Abstract

**Objectives:**

The aim of this study was to compare the difference in the level of clinical independence among predoctoral dental students during their community clinic rotation with their dental school clinic rotations.

**Methods:**

Descriptive statistics, assessment of sample normality, and *T*‐tests were performed to present the difference in average independence scale scores for the participants in each of the clinical disciplines at dental school clinical rotations and community rotations. The relative impact of each community clinical site was assessed to compare scores assigned at varying locations by different faculty.

**Results:**

This study analyzed 222 dental students, including 29 from International Dental Program (IDP) and 193 traditional Doctor of Dental Surgery (DDS) students. Community clinic scores showed greater variability, with significantly higher scores in diagnostics and operative dentistry compared to school clinics, where higher scores were noted in endodontics and oral surgery. IDP students scored lower than traditional DDS students in school clinics but outperformed them in community settings. Community clinic scores showed greater variation between 2023 and 2024 compared to the smaller changes observed in dental school clinic scores.

**Conclusions:**

This study highlights the impact of clinical setting, and duration on student procedural experience and performance, suggesting that varied clinical settings can enhance students' skills and readiness for practice. It reveals differences in DDS and IDP students' experiences due to prior training and potential evaluation biases. Study findings suggested lack of significant variance in the overall scores across different evaluators in community rotations. Future research should focus on refining evaluation metrics and better prepare students for practice.

## 1 INTRODUCTION

The implementation of community rotations in dental school curricula throughout the United States has been used to enhance the experiential learning of predoctoral dental students by integrating didactic teaching and preclinical training to public health settings.^1^ In fact, the Commission on Dental Accreditation states that dental education programs must make available opportunities and encourage students to engage in service and/or community‐based learning experiences.^2^ Integrating community‐based education into dental school curricula not only allows for the clinical application of comprehensive dental and oral healthcare knowledge, but also the potential to address healthcare equity issues such as inaccessibility, and disparities in population health.^3^, ^4^ Dental student participation in immersive rotations in nonmetropolitan areas has been demonstrated to positively impact the professional development of graduates.^3^, ^4^, ^5^ Community rotation experiences have provided further opportunities for predoctoral students to develop and improve upon their clinical expertise beyond structured school settings when it comes to applying effective communication styles, critical thinking, clinical reasoning and management, professionalism, and self‐awareness.^5^, ^6^

To effectively evaluate the impact of these community rotations on students’ level of independence in clinical settings, it is imperative to measure their skill level through assessable metrics, and make any modifications deemed necessary. Some dental schools have utilized a self‐assessment model, allowing students to evaluate their abilities prior to and after engaging in community‐based clinical experiences via pre‐ and postsurveys, reflective essay writing, and questionnaires.^6^, ^7^, ^8^ There have been various studies with respective tools developed to assess the impact of such experiences on improving social and behavioral skills like cultural competence,^9^, ^10^ while these factors are of importance, there are few studies that evaluate these rotations’ impact on the procedural clinical independence level of dental students, which is integral in effectively assessing the growth and development of the successive dental workforce. In dental education, it is crucial to compare assessment scores given by faculty in different environments because the evaluation of these skills can differ between assessors and impact the determination of competency.^11^ Additionally, comparing scores across diverse settings using both quantitative and qualitative measures ensures assessments accurately reflect the developing skills and competencies essential for clinical practice.^12^ This comprehensive assessment system is pivotal not just for evaluating trainees' advancement but also for shaping educational programs. Evaluating assessment scores across these settings ensures that graduates possess the essential skills and competencies to succeed in diverse professional environments, promoting their long‐term expertise, success, and adaptability.^13^, ^14^

Since the 1970s, dental schools have offered modified predoctoral programs for foreign‐trained dentists (FTDs), with 41 out of 71 schools currently providing International Dental Programs (IDPs) that enroll approximately 600 FTDs each year.^15^, ^16^, ^17^ The United States is the leading destination for international students seeking advanced education and research opportunities.^18^

Understanding the differences in preparedness between traditional Doctor of Dental Surgery (DDS) students, who typically follow a 4‐year curriculum, and FTDs, in accelerated or condensed programs, is essential for assessing overall clinical proficiency.^15^, ^17^ International dental students often have prior dental educational experience, that may differ significantly from US standards, influencing their didactic and clinical performance due to distinct curricula and teaching methods at non‐US institutions.^15^, ^19^, ^20^ While most FTDs report improved knowledge and clinical skills after completing their programs, there is limited evidence on the educational outcomes of these programs.^19^, ^20^ The Indiana University School of Dentistry (IUSD) offers a full‐time IDP that spans over 30 months.^21^

In 2020, IUSD adapted and implemented a modified Ottawa scale,^22^, ^23^ for daily clinical assessment at dental school clinics. The Ottawa scale (O‐SCORE) was validated by comparing it with well‐established assessment tools, such as the Objective Structured Assessment of Technical Skills (OSATS) checklist and OSATS Global Rating Scale (GRS), to evaluate its reliability and minimize biases like end‐aversion.^23^, ^24^ Generalizability analysis showed that training level accounted for most of the score variance, clearly distinguishing between competency levels, from novice (PGY‐1) to experienced staff surgeons.^24^ Post hoc tests confirmed the scale's ability to differentiate between groups.^24^ The final item, assessing trainees' readiness for independent procedures, revealed significant differences between those prepared for independent practice and those who were not.^24^ With a high reliability coefficient and strong agreement between assessors, the O‐SCORE consistently provided reliable and valid ratings across various proficiency levels.^24^

The Ottawa independence scale allows faculty to more reliably measure student performance based on how much guidance a student needs during each dental procedure. It assesses performance at each patient encounter, concentrating on the student's preparedness for independent practice.^25^ By prioritizing daily performance over summative evaluations, the scale encourages a more comprehensive discussion about clinical performance. Furthermore, it monitors student advancement toward autonomy over time, allowing educators to focus on areas where the students may require more efforts to improve their independence in practice.^25^ This assessment tool was developed and adapted to capture a large sample of individual assessments which, upon aggregation, provide more reliable predictors of future clinical performance of predoctoral students and allow faculty to make better entrustment decisions with the goal of preparing graduates for safe, independent practice without supervision.^22^, ^26^, ^27^

The Community‐Based Dental Education (CBDE) program at IUSD was developed and implemented with support from Health Resources Services Administration (HRSA‐18‐014) in September of 2018. The program has been instrumental in developing strong community partnerships through successful implementation of robust, immersive, community rotations for fourth‐year dental students at federally qualified health centers (FQHCs), and similar settings within the dental safety net. Initially, when the program was launched in 2018, the rotation lasted for 2 weeks at FQHCs. However, beginning in 2023 (for the class of 2024), the rotation has been extended to include 3 weeks at FQHCs and an additional 1 week at a private practice. All predoctoral students at IUSD must complete the community rotation for 3 weeks in their fourth year of dental school at an affiliated community site under the supervision of a licensed community site dentist. The CBDE program uses the same independence scale as the dental school clinics to rate the students' level of clinical independence during their community rotations.

This study compares the difference in the level of clinical independence scores among predoctoral dental students during their dental school's clinical rotations and their community site rotation. Additionally, it compares these scores between students in the traditional DDS program and those in the IDP. This study also includes comparison of these scores between two class cohorts. This is the first study that we know of that aims to use an independence scale for daily clinical assessment to gauge students’ readiness for practice during community rotations.

## 2 METHODOLOGY

This study was approved as exempt by Indiana University's Institutional Review Board (Protocol #14692). This study was a retrospective review of student records where the data provided to the research team were completely anonymized, hence did not require informed consent. The anonymized data were accessed in April 2024.

This cross‐sectional, retrospective study compares the difference in the level of clinical independence among predoctoral dental students during their dental school's clinical rotations and their CBDE community site rotation.

**FIGURE 1:**
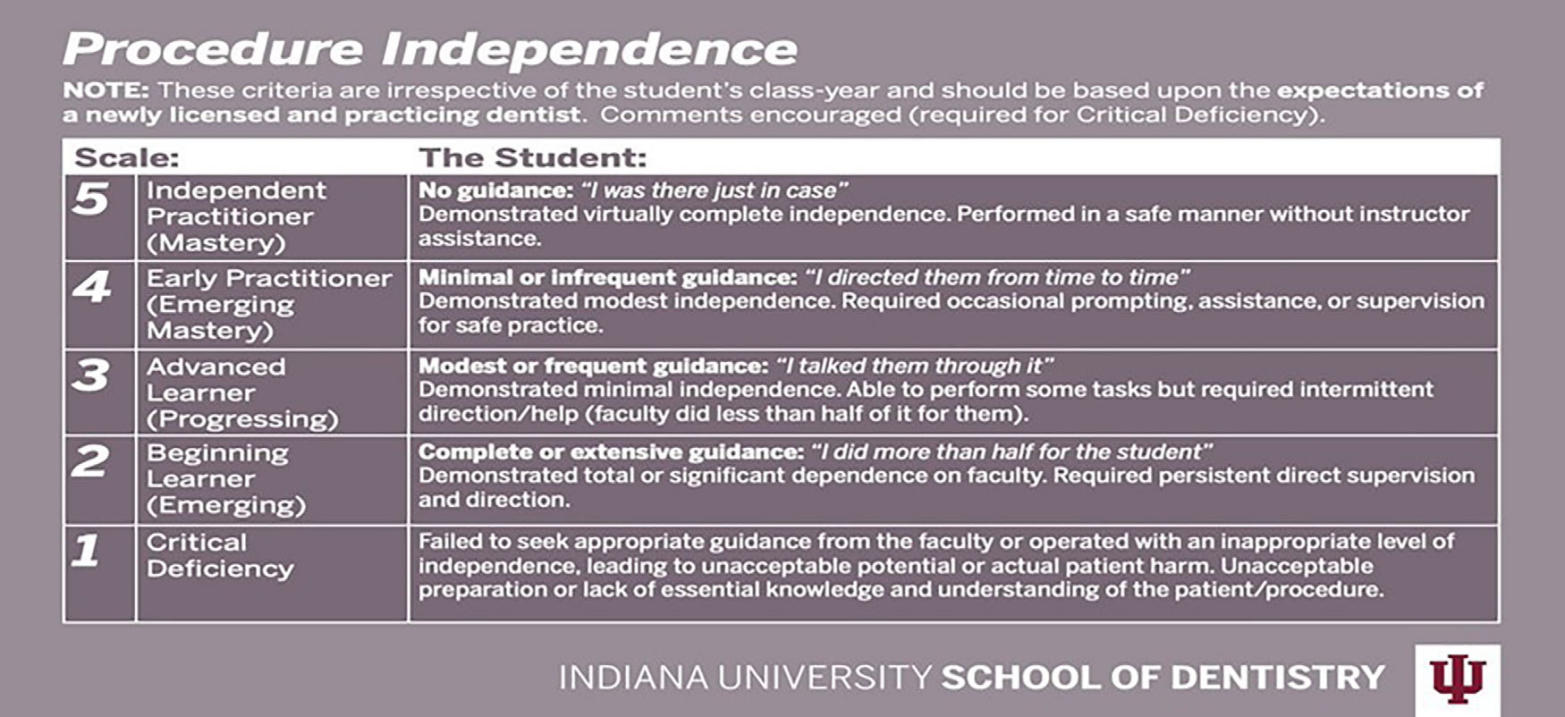
Independence scale.

The Ottawa independence scale measures how much guidance a student needs using a defined rubric (Figure 1) including the following possible scores: 1—Critical Deficiency; 2–Beginning Learner (Complete or Extensive Guidance); 3—Advanced Learner (Modest or Frequent Guidance); 4—Early Practitioner (Minimal or Infrequent Guidance); and 5—Independent Practitioner (No Guidance). All attending clinical faculty of the predoctoral program at IUSD are calibrated on the independence scale through both didactic and in‐person sessions. In addition, the rubric for the independence grading scale is posted in each clinical operatory where the assessment takes place. Attending faculty make each assessment based on the expectations of a newly licensed and practicing dentist, which is then captured in the evaluation module of the electronic health record management system, Axium, at the end of each patient encounter.^28^ Custom Axium reports indicate the total number of procedures completed and the average values of individual assessments cumulatively and stratified by dental discipline across a defined time period, which more reliably predict future clinical performance.^29^

The calibration process for community site faculty is similar and includes a didactic component which involves reviewing modules that include various clinical disciplines: Some of the modules include—endodontic, periodontics, caries risk assessment, radiology prescription and interpretation, asepsis, Personal Protective Equipment and infection control, oral pathology, and silver diamine fluoride. At the end of each module, the faculty complete a quiz. Additionally, community site faculty are invited to an annual in‐person calibration meeting. This meeting updates them on the school's assessment standards including the clinical assessment tool that is used, any modifications or enhancements to the evaluation criteria, and outlines expectations for the community site faculty. The rubric for the independence scale is again shared before the beginning of student rotations for each year.

### 2.1 Inclusion criteria

**FIGURE 2:**
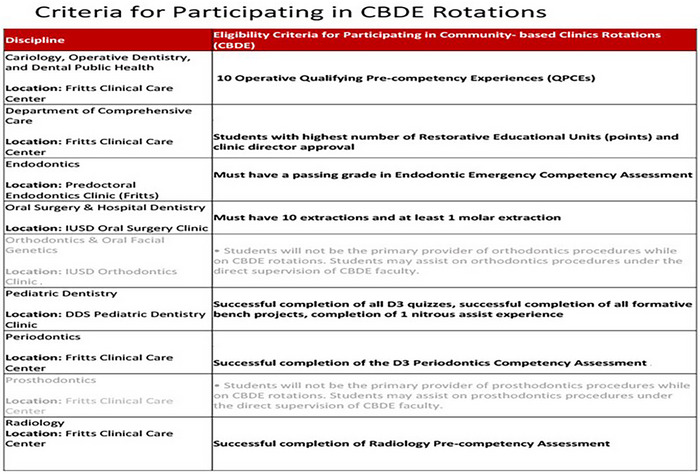
Predoctoral student eligibility criteria for community‐based dental education (CBDE) participation.

This study represented two cohorts: 111 students in the class of 2023 (C2023) and 111 in the class of 2024 (C2024). The CBDE program in the predoctoral curriculum is designed to provide an experiential clinical learning experience for fourth‐year students deemed eligible upon completion of the requirements (Figure 2). This eligibility criteria minimize bias as it selects for students based on specific clinical and didactic experiences

### 2.2 Cohort adjustments

- Three students from the C2023 dataset, who graduated in 2024, were moved to the C2024 dataset to ensure accurate cohort representation.
- One student who repeated their D4 year and graduated in 2024, rather than the initially expected 2023, was identified. To maintain data consistency, this student's data were removed from the C2023 dataset, and only their C2024 scores were retained, reflecting the CBDE rotations that occurred during the C2024 academic year.
- Students not included in the CBDE dataset were removed from the final analysis to ensure equal representation of students in both CBDE and clinic data.

### 2.3 Data extraction

For the analysis of clinical performance scores, data cleaning was performed on the provided datasets. Student names were anonymized, and university ID numbers were replaced. The independence scale assessment report for each student (linked to their student ID) for academic years 2023 and 2024 was retrieved from the Axium database by a school personnel compliant under the Family Educational Rights and Privacy Act (FERPA) law.^30^ The students’ community site rotations’ scores were also retrieved and the student's anonymity maintained. Students’ characteristics including demographic information (i.e., age, gender), as well as any additional clinical training acquired before enrolling in the DDS program (i.e., participants of International Dentist Program at IUSD)^31^ was obtained from school records and linked to their student ID. The linked information was deidentified and made available to the research team for analysis and interpretation.

### 2.4 Data analysis

Descriptive statistics were first performed to summarize the demographics of the students. Following this, a *t*‐test analysis was conducted to compare the mean clinical performance scores between the school clinics and community clinics (Table 1).

**TABLE 1:** Student demographics.

Variable	Categories	Count (*n*, percent of total)^a^
**Student type**	International Dental Program students	(*n* = 29, 13.06%)
	Traditional students	(*n* = 193, 86.94%)
**Age group**	20–29	(*n* = 156, 69.6%)
	30–39	(*n* = 58, 25.8%)
	40–49	(*n* = 10, 4.46%)
**Gender**	Female	(*n* = 133, 58.8%)
	Male	(*n* = 93, 41.1%)
**Race**	White	(*n* = 138, 61.6%)
	Asian	(*n* = 56, 25%)
	Black or African American	(*n* = 8, 3.5%)
	Native Hawaiian or other Pacific Islander	(*n* = 1, 0.4%)
	Hispanic	(*n* = 3, 1.3%)
	Multicultural/multiracial	(*n* = 8, 3.5%)
	Other	(*n* = 10, 4.46%)

^a^ Count (percent of total).

The comparative analysis included disciplines for which students received scores from both the dental school clinic and community clinic site faculty. These disciplines included diagnostics, preventive dentistry, periodontics, oral surgery, endodontics, operative dentistry, and pediatric dentistry. Additionally, a comparison was made between the mean scores of IDP students and Traditional DDS (non‐IDP) dental students across the aforementioned disciplines in the two settings, using *T*‐test to identify any statistically significant differences in performance between the groups.

A preliminary power analysis was conducted prior to the main statistical tests to ensure that the sample sizes were comparable to detect statistically meaningful effects. This step was crucial for determining the minimum number of participants required in each group to achieve adequate statistical power. Assuming unequal variances due to the differences in evaluators between the clinic and CBDE faculty, a two‐sample *t*‐test was employed for the analysis. The null hypothesis (H0) was that the mean scores for the clinic and CBDE were equal (*μ*1 = *μ*2). The significance level (*α*) for the test was set at 0.05, and a two‐sided test was used to account for differences.

Furthermore, a year‐by‐year comparison of mean scores across various disciplines was carried out between the class of 2023 and the class of 2024.

Additionally, the relative impact of a community site on the overall CBDE mean was evaluated by calculating the percent contribution of each evaluator to the overall CBDE mean score.

## 3 RESULTS

The student body comprises IDP students (*n* = 29), who were trained as foreign dentists prior to enrolling at IUSD, representing 13.06% of the total, and traditional DDS students (*n* = 193), accounting for 86.94%. In terms of age distribution, students aged 20–29 years (*n* = 156) constituted 69.6% of the population; Gender distribution showed females (*n* = 133) comprising 58.8% of the population with Caucasian students (*n* = 138) making up 61.6% of the cohort, followed by Asian students (*n* = 56; 25%) as illustrated in Table 1.

At school clinics, the top three procedures were diagnostic, (*n* = 24,414; 38.05%), followed by operative procedures (*n* = 17,688; 27.88%), and prosthodontic procedures (*n* = 8719; 3.74%). In contrast, at community clinics, the most frequently performed procedures were operative, with 4135 procedures making up 44.5% of the total, followed by diagnostic (*n* = 2234; 24.5%), and oral surgery procedures (*n* = 1150; 12.3%).

Notable differences in other procedures include a higher proportion of preventative care, endodontic and periodontic procedures, at school clinics, while pedodontics' procedures were more offered at community clinics. Additionally, Orthodontics procedures were only recorded at school clinics. The power analysis established the following sample sizes: for *t*‐tests with a moderate effect size (0.5), 64 participants were required per group. The results of the power analysis are included in Appendix I.

Table 2 illustrates the variety of procedures completed by students at both school clinics and community clinics. Higher evaluation scores for students at community clinics as compared to dental school clinics were observed in the diagnostics (4.47 vs. 4.22) and operative dentistry (4.33 vs. 4.18) disciplines, with these differences being statistically significant (*p* < 0.05). Conversely, students received higher scores at school clinics in endodontics (4.43 vs. 3.97) and oral surgery (4.32 vs. 4.02), with statistically significant differences (*p* < 0.05). In pediatric dentistry (4.24 vs. 4.15) and periodontics (4.42 vs. 4.35), students' scores were slightly higher at school clinics, but the differences were not statistically significant (*p* > 0.05), indicating comparable evaluations between the two settings.

**TABLE 2:** Clinical procedures by discipline: school clinics and community clinics.

Discipline	Dental school clinics *n* (%)	Community clinics *n* (%)
*Diagnosis*	24,414 (38.05)	2234 (24.5)
*Endodontics*	984 (1.55)	160 (1.72)
*Orthodontics*	153 (0.24)	N/A
*Oral surgery*	3185 (5.02)	1150 (12.3)
*Periodontics*	1503 (2.37)	22 (0.2)
*Preventative*	4714 (7.43)	211 (0.022)
*Prosthodontics*	8719 (13.74)	276 (0.029)
*Operative*	17,688 (27.88)	4135 (44.5)
*Pedodontics*	2086 (3.29)	1100 (11.8)
**Total**	63,446	9288

In the comparison of IDP students and traditional DDS students across dental school clinics and community clinics, several key differences emerged. In dental school clinics, IDP students scored lower than traditional DDS students in diagnostic (4.13 vs. 4.24, *p* < 0.05), pediatric dentistry (4.13 vs. 4.25, *p* < 0.05), and operative dentistry (4.15 vs. 4.19, *p* < 0.05). The overall mean score also showed a significant difference, with IDP students scoring lower than traditional DDS students (4.18 vs. 4.25, *p* < 0.05). In community clinics, IDP students had significantly higher scores than traditional students in diagnostic (4.59 vs. 4.45, *p* < 0.05) and demonstrated a higher overall mean score compared to traditional DDS students (4.50 vs. 4.32, *p* < 0.05). Table 3 presents student mean scores across dental disciplines and the results from two‐sample *t*‐tests comparing mean scores between school clinics and community clinics, as well as between IDP students and traditional DDS students.

**TABLE 3:** Discipline mean scores and two‐sample *t*‐test: clinic versus community clinics, and International Dental Program (IDP) versus traditional Doctor of Dental Surgery (DDS) students (non‐IDP).

Discipline	Dental school clinic mean	Community clinic mean	*p* value	IDP^a^ dental school clinic mean	Non‐IDP^b^ dental school clinic mean	*p* value	IDP^a^ community clinic mean	Non‐IDP^b^ community clinic mean	*p* value
Diagnostic	4.22	4.47	<2.2e‐16 (*)	4.13	4.24	<2.2e‐16 (*)	4.59	4.45	0.0319 (*)
Endodontics	4.43	3.97	0.0004 (*)	4.43	4.44	0.9020	4.25	3.94	0.4162
Oral surgery	4.32	4.02	1.534e‐8 (*)	4.27	4.32	0.1437	4.20	4.00	0.2027
Pedodontics	4.24	4.15	0.1555	4.13	4.25	0.0040 (*)	4.42	4.11	0.0907
Periodontics	4.42	4.35	0.2772	4.41	4.42	0.8822	4.50	4.32	0.3470
Operative	4.18	4.33	0.0002 (*)	4.15	4.19	0.0029 (*)	4.45	4.31	0.2063
Overall	4.24	4.34	6.766e‐14 (*)	4.18	4.25	<2.2e‐16 (*)	4.50	4.32	4.233e‐6 (*)

^a^ International Dental Program.

^b^ Non‐International Dental Program (traditional DDS students).

* *p* < 0.05.

**FIGURE 3:**
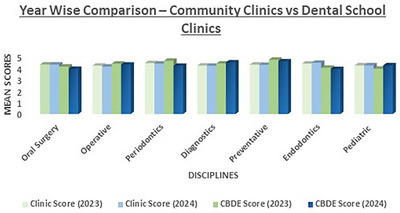
Year wise comparison—community clinics versus dental school clinics.

When comparing school clinic scores to community clinic scores between 2023 and 2024, significant trends were observed in the comparison of dental school and community clinic scores. School clinic scores for operative dentistry showed a significant decrease, dropping from 4.22 to 4.14. The community clinic scores experienced substantial decline in scores in several disciplines: preventative (4.73 vs. 4.59), oral surgery reduced from (4.14 vs. 3.94), and endodontics (4.03 vs. 3.92). Overall, community clinic scores displayed more pronounced variations compared to the relatively smaller changes observed in dental school clinic scores during this period. The results are detailed in Figure 3.

The results from relative impact of evaluator‐specific scores on overall mean indicated no significant variance, and no outliers were observed (Table 4). The overall mean impact of each community site is considered low to moderate, and no evaluators were found to significantly skew the results. Out of the 26 faculty evaluators across different sites, the overall CBDE score mean for all disciplines and classes was 4.34 ± 0.71. Particularly, Evaluator F13 evaluated only one student, assigning a mean score of 3.6, while Evaluator F19 scored the same student with a mean of 4. Additionally, Evaluator F26 gave scores other than 5 on just four occasions out of 118 evaluations in total.

**TABLE 4:** Evaluators assessment: mean scores and relative impact.

Evaluator	Number category evaluations	Mean	Relative impact
F13	10	3.6	10.59%
F16	85	3.66	9.74%
F26	118	4.96	8.94%
F18	256	4.84	7.19%
F21	18	4.83	7.16%
F11	57	3.88	6.60%
F5	177	4.76	6.14%
F22	62	4.73	5.61%
F9	166	4.69	5.05%
F19	800	3.99	4.94%
F14	50	4.66	4.66%
F6	238	4.03	4.41%
F2	25	4.64	4.41%
F25	77	4.57	3.39%
F3	147	4.18	2.29%
F4	34	4.47	1.94%
F20	44	4.43	1.38%
F15	43	4.26	1.15%
F8	159	4.40	0.87%
F10	99	4.39	0.83%
F1	17	4.39	0.78%
F12	53	4.28	0.76%
F17	67	4.48	0.75%
F7	117	4.31	0.41%

## 4 DISCUSSION

Previous studies have shown that community rotations during dental school training have led to an increased understanding and improvement of clinical competence, access to care, interprofessional experience, and community engagement among predoctoral dental students.^32^, ^33^ Literature shows, participating students were able to perform more number of dental procedures in less time with similar or increased revenue equivalents,^34^, ^35^ a variety of procedures, and get better patient care experience at community rotations.^4^, ^34^, ^36^, ^37^ Thus, well‐constructed community experiences have the potential to positively influence aspiring providers and address community needs through service‐learning partnerships, creating a mutually beneficial model for clinicians, patients, and stakeholders alike. These experiences also play a significant role in improving students’ ability to provide care as independent practitioners inside and outside institutional settings.

A study on the impact of externship length at Boston University School of Dental Medicine found that longer externships (10 weeks) allowed students to perform more complex procedures and more procedures per week compared to shorter externships (6 weeks).^38^ This implies that extending the duration of community rotations might similarly boost students' clinical experience, efficiency, and proficiency, consistent with the greater procedural output and complexity observed in longer externships. Our study results, however, contrasted with the above findings, with students who participated in the community rotations for 3 weeks scoring lower than those that participated for 2 weeks. It must however be noted that it involved different faculty supervising students during the additional week. The program acquired new community partners (private offices) in the year 2024 that offered 1 week rotation, in addition to the initial 2 weeks rotation at the FQHCs. A week‐long rotation gives much lesser time for students to get accustomed to a new surrounding which may have led to poorer scores, bringing down their mean scores for that year. Another point worth noting is these comparisons are based on average scores from dental school clinics where the students spend close to one full year whereas, the community rotations last only 3 weeks.

In our study, the higher mean scores in the disciplines of diagnostics and operative dentistry at community clinics are likely due to greater number of procedures such as oral evaluations, x‐rays, and direct restorations being offered at these clinics, as noted by previous studies.^34^, ^35^ Students achieved higher average scores in endodontics and oral surgery at school clinics compared to community clinics, likely because many community sites refer more complex procedures to specialists and may not offer adequate evaluation due to limited community site faculty scope in assessing these disciplines. Medicaid reimbursement coverage is another factor limiting the type of procedures that can be offered in these settings. In Indiana, the Healthy Indiana Plan (HIP) Basic (coverage program under Indiana's Section 1115 waiver) does not cover extensive dental services primarily covering oral exams and x‐rays, but those who qualify for the HIP plus program receive comprehensive dental benefits, including^39^, ^40^ bitewing x‐ray series annually, and a full‐mouth or panoramic radiograph once every 5 years. The plan also covers restorative services such as fillings and extractions (up to four per year), and one crown per benefit year.^41^

Our study showed that scores in community clinics exhibited greater variability across different disciplines compared to the more consistent scores observed in dental school clinics during the same period. This variability could be attributed to the fact that community clinics often serve a more diverse patient population such as treating underserved patients with varying needs, providing students with a broader range of clinical scenarios, unique learning opportunities, and real‐world challenges.^33^, ^42^ This diversity may lead to greater fluctuations in student performance as they adapt to different environments and patient interactions. However, further analysis revealed that the impact of evaluator‐specific scores on the overall mean was minimal, with no significant differences observed among the scores of various community site faculty. This consistency indicates that the scoring system is reliable across different settings. The absence of significant outliers or deviations from any individual evaluator can be attributed to the internal consistency of the evaluation criteria employed, suggesting that the standardized calibration process implemented across various community sites as a part of the CBDE program effectively minimized potential discrepancies, resulting in a fair and uniform evaluation system. Clinical expectations and the skills of the students evaluated justify any scores that, at initial evaluation, appear outside the norm. Similarly, a study examining the relationship between student evaluations, teaching effectiveness, and academic performance reported low variance in evaluations across different instructors, highlighting that consistent evaluation frameworks play a crucial role in ensuring fair assessment outcomes.^43^

Our study indicated that IDP students had significantly lower scores than traditional DDS students in diagnostic sciences, pediatric dentistry, and operative dentistry within dental school clinics, resulting in a lower overall mean score, but they outperformed in diagnostics and achieved a higher overall mean score in CBDE community clinics. One reason for these lower scores across various disciplines at dental school clinics could be the variability in program length and curriculum structure for FTDs.^44^, ^45^ Unlike traditional programs that have a set duration, IDP programs vary from 24 to 48 months, with an average duration of approximately 28 months.^44^, ^45^ This condensed timeframe may limit IDP students' clinical experience and exposure to diverse scenarios, potentially contributing to their lower scores in specific dental domains. Additionally, the differences in materials and procedures that IDP students learned in their home countries.^46^ Variations in educational quality across countries may lead to different levels of preparation and familiarity with techniques emphasized in US dental schools, potentially contributing to their lower scores. Another factor could be unconscious biases held by faculty in dental school clinics toward IDP students due to their prior training, leading to higher expectations and stricter grading.^47^ This may have resulted in lower scores for IDP students compared to traditional students. In contrast, community clinics, where faculty may not be aware of students' IDP status, offer more impartial evaluations based solely on performance. In contrast to our results, another study comparing the clinical experiences of FTDs in an Advanced Standing DMD Dental Program (DMDAS) with domestic dental students (DMD) showed that, by the end of their final year, both groups had comparable clinical experiences, despite DMDAS students having less time in school clinics.^48^ On average, DMD students completed greater number of clinical procedures than their DMDAS peers, particularly diagnostic procedures, direct restorations, periodontics, and removable partial dentures.^48^

Our study illustrates that the comparison between the Class of 2023 and the Class of 2024 shows stable dental school clinic scores with minor changes, while community clinic scores for the Class of 2024 experienced notable declines despite an extended rotation period. The class of 2024, being the first cohort to experience the extended rotation, may have faced challenges in adapting to the new schedule and expectations, particularly if they were not adequately prepared for the increased demands of an additional week in the community setting.^49^

### 4.1 Strengths and limitations

Some of the limitations in this cross‐sectional study involved level of generalizability. Additionally, factors related to externship site settings, such as variability in clinical operations, level of supervision, patient population, and differences in learning opportunities offered to students could have influenced the independence scale scores. Moreover, offering the same weight in the scale to less common procedures like endodontic and periodontic procedures, as the more common diagnostic and restorative procedures may have skewed our overall mean scores. Finally, this methodology relies on faculty calibration which is another weakness affecting the study methodology.

This study provided a unique analysis of dental procedures by discipline and compared it with their performance during community rotation that provides students diverse opportunities to enhance and improve upon technique sensitive skills that they may not have exposure to in conventional clinical settings.

Further studies should look at specific evaluation metrics to assess students’ clinical skills in dental education and study the role community rotations could play in formative assessments.

## 5 CONCLUSION

This study sheds light on the differing experiences of predoctoral dental students in both school‐based and community clinic settings, revealing the significant impact of varied clinical environments on student development. It emphasizes the benefits of community rotations in fostering greater independence in practice. The comparison between traditional DDS and IDP students highlights challenges such as differing prior training, program structure, and the potential influence of biases on evaluations. These insights affirm the need for continued refinement of clinical training programs to better prepare all students for independent practice, with particular attention to ensuring fair evaluations across diverse educational backgrounds. Future research should focus on optimizing assessment methods and understanding the full scope of factors influencing student readiness for practice.

## APPENDIX 1

### 1.1

Power analysis results for two‐sample *t*‐test.

**Table:**

Effect size	Recommended *n* per group
0.2 (Small)	**393**
0.5 (Moderate)	**64**
0.8 (Large)	**26**

Effect size: 0.5 (moderate); alpha: 0.05 (significance level: Type I error); power: 0.80 (desired power: 1 – Type II error).
